# Processes Underlying the Nutritional Programming of Embryonic Development by Iron Deficiency in the Rat

**DOI:** 10.1371/journal.pone.0048133

**Published:** 2012-10-26

**Authors:** Angelina Swali, Sarah McMullen, Helen Hayes, Lorraine Gambling, Harry J. McArdle, Simon C. Langley-Evans

**Affiliations:** 1 School of Biosciences, University of Nottingham, Sutton Bonington, Loughborough, United Kingdom; 2 Rowett Institute of Nutrition and Health, University of Aberdeen, Aberdeen, United Kingdom; The University of Manchester, United Kingdom

## Abstract

Poor iron status is a global health issue, affecting two thirds of the world population to some degree. It is a particular problem among pregnant women, in both developed and developing countries. Feeding pregnant rats a diet deficient in iron is associated with both hypertension and reduced nephron endowment in adult male offspring. However, the mechanistic pathway leading from iron deficiency to fetal kidney development remains elusive. This study aimed to establish the underlying processes associated with iron deficiency by assessing gene and protein expression changes in the rat embryo, focussing on the responses occurring at the time of the nutritional insult. Analysis of microarray data showed that iron deficiency *in utero* resulted in the significant up-regulation of 979 genes and down-regulation of 1545 genes in male rat embryos (d13). Affected processes associated with these genes included the initiation of mitosis, BAD-mediated apoptosis, the assembly of RNA polymerase II preinitiation complexes and WNT signalling. Proteomic analyses highlighted 7 proteins demonstrating significant up-regulation with iron deficiency and the down-regulation of 11 proteins. The main functions of these key proteins included cell proliferation, protein transport and folding, cytoskeletal remodelling and the proteasome complex. In line with our recent work, which identified the perturbation of the proteasome complex as a generalised response to *in utero* malnutrition, we propose that iron deficiency alone leads to a more specific failure in correct protein folding and transport. Such an imbalance in this delicate quality-control system can lead to cellular dysfunction and apoptosis. Therefore these findings offer an insight into the underlying mechanisms associated with the development of the embryo during conditions of poor iron status, and its health in adult life.

## Introduction

It is now well-established that early life exposure to an adverse nutritional environment can programme aspects of adult anatomy, physiology and metabolism and thereby determine risk of cardiovascular and metabolic disorders [1]–[3]. Evidence from retrospective cohort studies shows that anthropometric markers of sub-optimal maternal nutritional status during fetal development are predictive of cardiovascular disease, type-2 diabetes and chronic kidney disease [4], [5]. Experiments in animals that have manipulated either overall food supply or dietary composition such that one or more nutrients is limiting, strongly support the hypothesis that during nutritional stress, the fetus mounts adaptive responses in order to preserve growth or survival [6]–[11]. In the long-term the modifications to organ structure, hormone responsiveness or gene expression, which characterise the adaptation to stress, predispose to disease in later life.

On a global scale, iron deficiency is the most prevalent manifestation of malnutrition. Poor maternal iron status is a recognised risk factor for preterm delivery, low birthweight and neonatal death, particularly in developing countries [12], [13]. Iron deficiency anaemia is highly prevalent in many populations and in some parts of the world more than half of pregnant women will be affected. A number of rodent studies indicate that the imposition of iron deficiency prior to and during pregnancy has long-term effects upon the resulting offspring, with reports of high blood pressure [11], [14]–[16], impaired nephrogenesis [17] and altered glucose handling [16].

The mechanistic basis of this nutritional programming of disease has not been fully explored. Much of the research focus has been upon postnatal events and the progression from what is usually a low birthweight phenotype, to adult pathology. One of the outcomes of such work has been the observation that undernutrition frequently results in a remodelling of tissue structure [18]–[21]. For example, in the kidney, diverse nutritional insults including maternal protein restriction, food restriction and iron deficiency, lead to a lower nephron number in the offspring [22]–[24]. This, arguably, may be a driver of the renal and cardiovascular outcomes which are also associated with nutritional insult. Such studies do not, however, capture the primary response to undernutrition or explain how tissue morphology is programmed by the maternal diet. Studies of specific candidate genes suggest that there may be modifications to the epigenome which establish altered patterns of gene expression [25]–[27]. However, to date, there is no convincing evidence that these epigenetic modifications are widespread, or that they modulate expression of genes of relevance to renal or cardiovascular disease.

We have previously hypothesised that nutritional programming of physiological function occurs through changes in a limited number of “gatekeeper” processes [24]. These are genes or gene pathways for which the adaptive response during development is common to all of nutritional insults. Earlier work in our laboratories considered the mechanistic basis of programming in two separate nutritional programming models (iron deficiency and protein restriction), across two rat strains. By comparing whole genome array and proteomics data across both models and strains, this work identified a number of putative gatekeeper genes, proteins and processes which may offer a generalised mechanism to explain the common phenotypes exhibited by offspring of pregnant rats exposed to diverse nutritional insults [24]. The experimental approach used in this previous study discarded gene or protein expression changes which only occurred in response to one of the dietary treatments or in one of the strains of rat, as the lack of replication across models suggested that these were not central mechanisms to the programming of a common phenotype. However, the existence of a common phenotype in response to different dietary insults is not universally accepted and it may be the case that the common endpoints observed are actually a non-specific consequence of different programming pathways. The wealth of data produced from each model within the original gatekeeper study is worthy of more in depth analysis for identification of diet specific mechanisms. The previously reported analysis was constrained by the low numbers of gene targets which were differentially expressed across two rat strains and two nutritional interventions. The data analysis reported in this further paper focuses on the response of the embryonic genome and proteome to maternal iron deficiency in the Rowett Hooded Lister rat and exploits a larger pool of differentially expressed genes for pathway analysis and increases confidence in the demonstrated role of maternal nutritional status as a regulator of cell cycle in the developing embryo. As before, any significant changes in gene and protein expression will not automatically suggest a causal relationship with the observed phenotypic changes such as increased blood pressure, but pathway analysis may shed light on the potential mechanisms at play early in the programming process.

## Materials and Methods

### Ethical Approval

All animal experiments were performed in the BioResources Unit of the University of Nottingham, under license from the United Kingdom Home Office in accordance with the 1986 Animals (Scientific Procedures) Act. The study was approved by the UK Home Office (Project Licence PPL40/2990) and University of Nottingham Ethics Committee (approval ID SLE/005/07).

### Animals

As previously reported [24], 16 female Rowett Hooded Lister (RHL) rats (Rowett Institute of Nutrition and Health, University of Aberdeen, UK) were subjected to a 12h light (08∶00–20∶00)-dark (20∶00–08∶00) cycle at a temperature of 20–22°C with *ad libitum* access to food and water. For 4 weeks prior to mating, half of the rats were fed a control iron (50 mg Fe/kg; FeC) diet and the other half an iron deficient diet (7.5 mg Fe/kg; FeD; [11]) to ensure depletion of iron stores during pregnancy. Iron-deficient diets were isocaloric relative to the control diet. Previous studies in our laboratories have shown that this protocol reproducibly reduces both maternal and fetal liver iron content by approximately 70% and 50% respectively [11], [28], [29] Cornock *et al*., unpublished observation). At a weight of approximately 180–200g, females were mated with stud males. After conception, determined by the presence of a semen plug on the cage floor, females were single-housed and remained on their pre-pregnancy diet. During pregnancy the animals were weighed and food intake was recorded daily. This protocol has been previously demonstrated to result in elevated systolic blood pressure and lower nephron number in the adult offspring [11], [24].On day 13 of gestation, pregnant females were culled by CO_2_ asphyxia and cervical dislocation. Individual embryos and placentas were harvested and RNA and protein prepared from a pool of all male embryos within a litter as described previously [24].

### Microarray

An Affymetrix Genetitan Rat 230 microarray was performed by Service XS. This array comprises over 31,000 probe sets, analysing over 30,000 transcripts and variants from over 28,000 well-substantiated rat genes, as well as positive and negative controls. Before the labelling process, the integrity of all RNA samples was further checked using the Agilent 2100 Bioanalyser. Output data were supplied as Affymetrix CEL files and loaded into Genespring (Agilent). Data were normalised to QC controls and samples assigned to the appropriate diet group. Data were loaded into MetaCore (GeneGo) to identify pathways affected by iron deficiency. All microarray data is MIAME compliant and the raw data has been deposited in ArrayExpress (accession number E-MTAB-664).

### Real-time PCR

Real-time PCR was performed on the same embryonic RNA sample that was prepared for microarray, to further explore some of the gene expression changes observed in the microarray analysis. RNA was reverse transcribed using Moloney murine leukemia virus (MMLV) reverse transcriptase (Promega) and real time PCR was performed using Probe Master Mix (Roche) and Taqman Rat Custom Expression Assays (ABI) on a Lightcycler 480 (Roche) in 384-well optical reaction plates. Expression values and linearity were determined using a cDNA standard curve. Data were normalised to total cDNA levels measured by Oligreen reagent (Invitrogen) at 80°C [30].

### Proteomics

Proteomic analysis was carried out by the Proteomics Section at the Rowett Institute of Nutrition and Health (University of Aberdeen). Protein samples were loaded onto sixteen 8–16% acrylamide gels to separate proteins in each embryo sample by isoelectric focussing in the first dimension (pI range 3–10) and SDS-PAGE in the second dimension. Gels were stained using the Colloidal Coommasie stain method, and imaged on a Bio-Rad GS800 scanning densitometer, followed by analysis using Progenesis SameSpots software. Each image was quality assessed before selection of a ‘master gel’, to which the other 15 gels were aligned. Gels were assigned to either the FeC or FeD group and any spots with differences in area and density between groups were identified by ANOVA. Spots of interest were hand-cut from the SDS-PAGE gels. In-gel digestion and trypsinisation of the cut spots was performed on a Proteome Works System, Mass PREP Station Robotic Handling System and extracted peptides were analysed on a nano LC-MS/MS system using Q-Trap. The total ion current data was searched against the MSDB database using the MASCOT search engine (Matrix Science) with the following search criteria: allowance of 0 or 1 missed cleavages; peptide mass tolerance of ±1.5 Da; fragment mass tolerance of ±1.5 Da, trypsin as digestion enzyme; carbamidomethyl fixed modification of cysteine; methionine oxidation as a variable modification; and charged state as 2^+^ and 3^+^. The highest MASCOT results were further selected for best matches with criteria of protein identifications having a Mascot score higher than 40 (threshold) and more than one peptide match. The identification was considered only with a combination of the highest Mascot score and maximum peptide coverage.

### Statistical Analysis

All data was analysed using the Statistical Package for Social Sciences (SPSS, Inc, Chicago, IL, Version 18.0). Differences between groups were assessed using an independent t-test, unless indicated otherwise in the text. Values are expressed as mean ± SEM. P<0.05 was considered as significant.

## Results

### Maternal Weight Gain and Reproductive Performance

There was no difference in the body weights of dams in the iron deficient and control iron groups either at the commencement of pregnancy or at the time of embryo collection (day 13 of pregnancy; **Table 1**). Weight gain and average daily food intake (calculated from food remaining in hopper) was similar between the two groups and iron intake was, as expected, 6-fold higher in the control group (P<0.001). While the number of male embryos collected did not differ with maternal diet, those fed an iron deficient diet produced significantly fewer embryos in total (P<0.03).

**Table 1:** Maternal weight gain, food intake and reproductive performance (FeC = control iron; FeD = iron deficient; BW = body weight; D13 = day 13 of pregnancy; NS = not significant).

	FeC	FeD	P
n	8	8	
BW at conception (g)	223.9±7.7	231.3±9.2	NS
BW at D13 (g)	285.5±5.7	295.2±8.9	NS
BW gain (g)	61.6±4.7	63.9±3.0	NS
Average food intake (g/day)	26.7±2.5	29.6±2.4	NS
Maternal iron intake (mg/day)	1.3±0.1	0.2±0.02	0.001
No. of embryos	14.4±0.8	11.3±0.6	0.03
No. of male embryos	6.4±0.9	5.1±0.2	NS

### Microarray

2524 genes were differentially regulated between control and iron deficient embryos (P<0.05). Of these, 979 genes were up-regulated with iron deficiency and 1545 genes were down-regulated. Despite the severity of the maternal insult, the magnitude of the expression changes was generally modest and the greatest expression changes were for sex-determining region Y-box 4 (*SOX4*; 2.34-fold up-regulation with iron deficiency; P<0.04) and microtubule-associated protein 1B (*Map1b*; 2.21-fold up-regulation with iron deficiency; P<0.02). The greatest down-regulations in gene expression occurred with myosin VC (*Myo5c*; 0.63-fold change; P<0.01) and S100 calcium-binding protein A6 (*S100a6*; 0.69-fold change; P<0.01). The 20 genes exhibiting the greatest fold-changes up or down in relation to control are listed in **Table 2**. A full list of differentially regulated genes is provided in **Table S1**.

**Table 2:** Genes showing the greatest increase or decrease in expression in FeD embryos (n = 8) relative to controls. FC = fold-change.

Accession Number	p-value	FC	Gene Symbol	Gene Title
BE115519	0.04	2.35	Sox4	SRY (sex determining region Y)-box 4
BG378086	0.02	2.21	Map1b	Microtubule-associated protein 1B
BE110516	0.02	1.97	RGD1563798	Similar to BC040823 protein
BE116127	0.00	1.92	RGD1564560	Similar to RCK
BF522283	0.04	1.91	Ppp2r4	Protein phosphatase 2A activator, regulatory subunit 4
BF562309	0.03	1.85	Ng35	Ng35 pseudogene
BI290608	0.02	1.77	Adipor2	Adiponectin receptor 2
BF562800	0.05	1.75	LOC683788	Similar to Fascin (Singed-like protein)
AF389425	0.02	1.73	Dpysl3	Dihydropyrimidinase-like 3
AI073272	0.05	1.69	Tor1b	Torsin family 1, member B
BF407469	0.02	1.67	Mbrl	Membralin
AI236185	0.03	1.63	Rcc2	Regulator of chromosome condensation 2
X74211	0.01	1.60	Map2	Microtubule-associated protein 2
BF389675	0.03	1.57	Cbx1	Chromobox homolog 1 (HP1 beta homolog Drosophila )
AI228656	0.03	1.56	Znf618	Zinc finger protein 618
BE111692	0.01	1.55	Myst3	MYST histone acetyltransferase (monocytic leukemia) 3
BF398680	0.02	1.52	Map4k4	Mitogen-activated protein 4 kinase 4
NM_021597	0.03	1.51	Eif2c2	Eukaryotic translation initiation factor 2C, 2
AW525560	0.02	1.50	LOC683788	Similar to Fascin (Singed-like protein)
L38247	0.05	1.48	Syt4	Synaptotagmin IV
BF420807	0.01	0.63	Myo5c	Myosin VC
AF140232	0.01	0.69	S100a6	S100 calcium binding protein A6
AA817746	0.03	0.70	LOC365985	Similar to adenylate kinase 5 isoform 1
NM_033234	0.03	0.72	Hbb	Hemoglobin, beta
AI179412	0.00	0.72	Frrs1	Ferric-chelate reductase 1
AW535082	0.00	0.73	Mthfs	5,10-methenyltetrahydrofolate synthetase (5-formyltetrahydrofolate cyclo-ligase)
BM390697	0.01	0.73	Gca	Grancalcin
AB001382	0.02	0.74	Spp1	Secreted phosphoprotein 1
AA819034	0.01	0.75	isg12(b)	Putative ISG12(b) protein
AI105366	0.00	0.75	Pyroxd2	Pyridine nucleotide-disulphide oxidoreductase domain 2
NM_020082	0.01	0.75	Rnase4	Ribonuclease, rnase A family 4
BI282268	0.04	0.75	Hdhd2	Haloacid dehalogenase-like hydrolase domain 2
NM_017134	0.01	0.75	Arg1	Arginase, liver
AI176041	0.01	0.76	Pir	Pirin (iron-binding nuclear protein)
AI029991	0.00	0.76	Clec1b	C-type lectin domain family 1, member b
AI548039	0.00	0.76	Nepn	Nephrocan
NM_080890	0.00	0.76	As3mt	Arsenic (+3 oxidation state) methyltransferase
BF284695	0.03	0.77	Wdr43	WD repeat domain 43
BF418169	0.00	0.77	Trex2	Three prime repair exonuclease 2
NM_020308	0.05	0.77	Adam15	A disintegrin and metallopeptidase domain 15 (metargidin)

### Pathway Analysis

**Figure 1:**
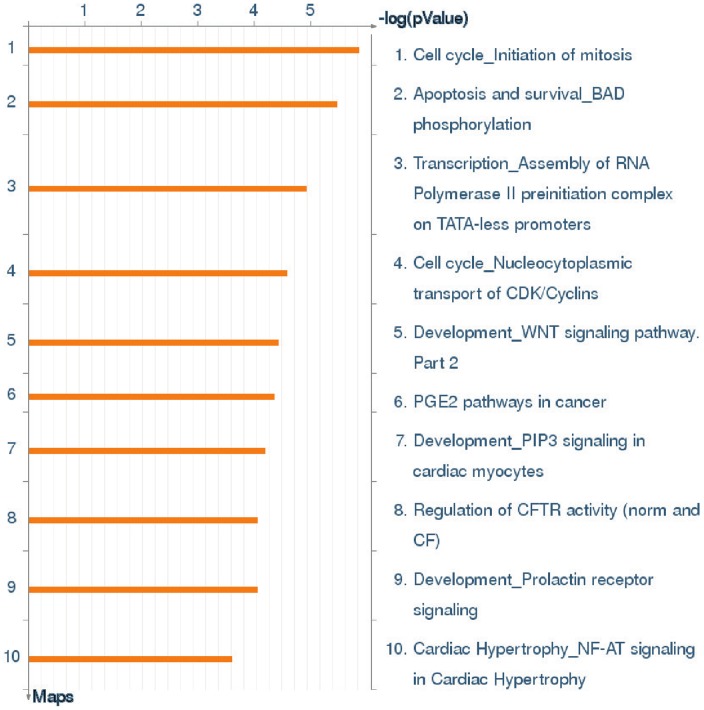
Significant GeneGo pathways affected by prenatal iron restriction in RHL rats. Processes are ranked based upon p-value (P<0.0003), bars represent inverse log of the p-value.

Pathway analysis was performed using the MetaCore platform to identify the functional processes associated with the significant gene expression changes following exposure of the embryo to maternal iron deficiency. The pathways which were most significantly affected (Figure 1; P<0.0003) were mostly due to down-regulation of associated genes. 19–42% of pathway genes were differentially regulated. These pathways were concerned with initiation of mitosis (associated genes: *cdk1, cdk7, ccnb1, ccnb2, ccnh, AP-2A, Mnat1, Akt, APC*; 36% of total pathway genes significantly regulated), BAD phosphorylation (*G-protein B, PDK, Akt, PKA, cDK1, JNK1, IRS-1, cytochrome c*; 26% of genes), assembly of RNA polymerase II preinitiation complex (*TFII-I, APP, PCNA, Mxi1, DHFR*; 38% of genes), nucleocytoplasmic transport of CDK/cyclins (42% of genes), WNT signalling (21% of genes), PGE2 in cancer (20% of genes), PIP3 signalling in cardiomyocytes (21% of genes), regulation of CFTR activity (19% of genes), prolactin receptor signalling (19% of genes) and NF-AT signalling in cardiac hypertrophy (25% of genes) (**Figure 1**). The pathway analyses identified a number of transcription factors which appeared to act as central “hubs”, or linkers, of other pathways and processes including organ development and cellular metabolic processes. *SP1* interacted with 303 network objects or genes which were differentially expressed in iron deficient embryos (Table S1) (e.g. *Pole-3, NCOA3*), *p53* with 102 (e.g. *cyclin H, Sumo-1)* and *c-myc* with 203 (e.g. *STAT3, Txn1*). The expression of these transcription factors was determined by real-time PCR (see below).

### Real-time PCR

**Figure 2:**
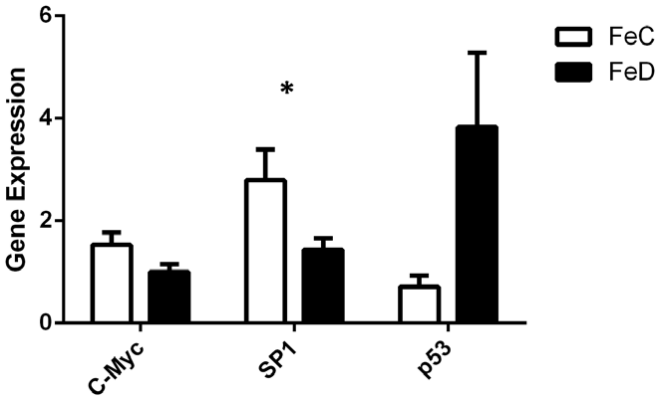
Transcription factor expression in embryos exposed to control (FeC) and iron deficient (FeD) diets, measured by RT-PCR and normalised with Oligreen; data shown as mean ± SE; n = 8; *P<0.05.

The expression of ten genes and the three transcription factors, identified by microarray analysis to be differentially expressed in the iron deficient embryo, was also confirmed using real-time PCR. In line with the key processes identified by the pathway analysis, these genes had a range of functions including regulation of cell growth and proliferation and cancer development (*PSAT1, Hint, SDCCAG10, p53 and C-myc*), cell cycle (*Ube2c, Hint, GMPS and p53*), apoptosis (*Hint*), protein synthesis and folding (*SDCCAG10, TOMM34, eEF1G*), cardiac muscle growth (*USMG5*) and developmental processes (*TBX3, FGFR1*). *SP1* and *c-myc* were down-regulated in embryonic tissue in response to maternal iron deficiency (**Figure 2**). The expression of *p53* was increased by more than 5-fold in the iron deficient embryos. These results are supported by findings that iron induces the expression of *c-myc* [31] and *SP1* [32], while inhibiting *p53* expression via the induction of *mdm2* [33]. The change in expression of these transcription factors would therefore have implications for the many downstream target genes identified by the pathway analysis (e.g. see above; *Pole-3:* FC 0.81, P<0.01; *NCOA3:* FC 1.19, P<0.01; *Cyclin H*: FC 0.93, P<0.03; *Sumo-1*: FC 0.89, P<0.001; *STAT3:* FC 1.25, P<0.007; *Txn1*: FC 0.94, P<0.03). The real-time PCR analysis of the gene targets identified in the microarray and pathway analysis generally supported the outcome of the microarray results (**Table 3**). Although the fold-changes identified by microarray were generally small (though highly significant), for seven out of the ten genes we showed good agreement between array and real-time PCR in terms of statistical significance and the direction of change in expression. Only *Hint, eEF1G* and *FGFR1* failed to show consistency between the two methods, as although significant differences in expression were noted between controls and iron deficient embryos, these genes were up-regulated by rtPCR compared to down-regulated in the arrays.

**Table 3:** Microarray and Real-time PCR determination of gene targets in whole embryonic tissue exposed to a maternal iron deficient diet, relative to a control iron diet (FC = Fold change); n = 8.

Gene	Accession No.	Microarray		PCR	
		P value	FC	P value	FC
TBX3	BE113656	0.009	0.76	0.03	0.75
SDCCAG10	BF416387	0.018	0.85	0.03	0.66
TOMM34	AI412736	0.015	0.87	0.03	0.69
PSAT1	AI230228	0.021	0.93	0.01	0.6
USMG5	AA891707	0.017	0.90	0.01	0.61
GMPS	BI283031	0.011	0.92	0.01	0.59
UBE2C	BI296084	0.039	0.94	0.001	0.45
HINT	AI227884	0.006	0.96	0.009	4.0
eEF1G	BM391203	0.014	0.95	0.08	2.50
FGFR1	BI275155	0.047	0.88	0.03	1.93

### Proteomics

Proteomics analysis identified 7 proteins which were significantly up-regulated in FeD embryos compared to FeC, and 11 proteins which were down-regulated with iron deficiency **(** **Table 4** **)**. Many of the identified proteins play important roles in cell proliferation (alpha-enolase, ADP-ribosylation factor-like 3, nucleophosmin, prohibitin, dihydrofolate reductase), protein transport and folding (ADP-ribosylation factor-like 3, nucleophosmin, vasolin-containing protein, Chaperonin containing TCP1, subunit 5), cytoskeletal remodelling (ADP-ribosylation factor-like 3, Chaperonin containing TCP1, subunit 5) and the proteasome complex (SUG1, Proteasome subunit alpha type 3-like).

**Table 4:** Proteins identified to be significantly differentially regulated by mass spectrometry following 2D gel electrophoresis.

Direction FeD to control	Protein	Function
Up	Alpha-enolase	Glycolysis; Cell proliferation
	Trypsin I precursor	Digestion
	Carbonyl reductase	Reduction catalyst
	ADP-ribosylation factor-like 3	Cell proliferation; Cytoskeletal remodelling; Protein trafficking
	Fructose-biphosphate aldolase A	Glycolysis, gluconeogenesis
	Farnesyl-pyrophosphate synthetase	Steroid/lipid synthesis
	SUG1	26S Proteasome subunit
Down	?ihydropyrimidinase-related protein 2	Cytoskeletal remodelling
	Chaperonin containing TCP1, subunit 5 (Epsilon)	Protein folding; Cytoskeletal remodelling
	Valosin-containing protein	Export of misfolded proteins
	Alpha-enolase	Glycolysis; Cell proliferation
	Phosphopyruvate hydratase	Glycolysis, gluconeogenesis
	Nucleophosmin	Protein chaperone; Cell proliferation
	Proteasome subunit alpha type 3-like	Proteasome complex
	Prohibitin	Protein chaperone; Cell proliferation
	Dihydrofolate reductase	Folate metabolism; Cell proliferation
	Transthyretin precursor	Thyroxine and retinol transport
	Dihydrolipoamide S-acetyltransferase	Pyruvate dehydrogenase complex

Dihydrofolate reductase (microarray: 0.88-fold change, P<0.001; proteomics: 0.8-fold change, P<0.04) and proteasome alpha-3 (microarray: 0.92-fold change, P<0.02; proteomics: 0.8-fold change, P<0.01) also showed significant changes in gene expression with the microarray, in the same direction as the proteomics analysis. In addition, Chaperonin containing TCP1, subunit 8, was also significantly down regulated in the microarray (0.9-fold change, P<0.004; subunit 5 protein: 0.8-fold change, P<0.004).

Alpha enolase was identified as both an up-regulated and down-regulated protein. This is probably due to the membrane translocation of the enzyme which is allowed through post-translational modifications such as acylation or phosphorylation [34].

## Discussion

The aim of this study was to explore the underlying mechanistic pathways which may explain the development of metabolic disease following *in utero* exposure to iron deficiency in a well-established rat model. We have previously suggested that a limited number of gatekeeper processes could explain the common phenotype which manifests in offspring of both prenatal iron and protein restriction in two different strains of rat [24]. However, the current study is much more specific and aimed to establish if additional, or alternative, diet-specific pathway responses occur with iron deficiency alone in RHL rats. Such responses may be associated with the reduced nephron endowment and increase in blood pressure previously found in this cohort [24] compared to the offspring of iron-replete controls, although this study did not set out to establish definitive causality.

This study was well powered to confidently identify differences in gene and protein expression between groups, and in excess of 2500 embryonic genes were found to be differentially expressed with maternal iron deficiency compared to exposure to a control diet. This offered a much greater and varied pool of genes to work with than the original gatekeeper study, which only considered the 153 gene changes reflected by both diets, or both strains. Pathway analysis of microarray data allows this mass of data to be refined so that the specific *processes* affected by maternal iron deficiency can be identified. In the previous gatekeeper study [24], aspects of cell cycle regulation represented four of the seven most significant pathways. It is noteworthy that the pathway most significantly impacted by iron deficiency in RHL embryos was the initiation of mitosis. This to some extent confirms the findings of our earlier study [24]. Specific genes affected in this pathway included *cdk1, cdk7, Cyclin B1, Cyclin B2 and Cyclin H.*

*Cdk1* and *cyclin B* form a complex initiating the onset of mitosis, shuttling back and forth between the nucleus and cytoplasm. Their gene expression, the initiation of mitosis and nucleocytoplasmic transport of *cdk* and *cyclins* were significantly affected by iron deficiency. Phosphorylation of the BH3-only protein BAD was the second most impacted process with iron deficiency. This is important during development when neuron numbers are controlled to ensure the correct architecture of the nervous system (Konishi et al, 2002). Iron deficiency specifically affected the WNT signalling pathway’s role in development. During embryogenesis, WNT proteins are involved in regulation of cell fate and patterning.

The two genes demonstrating the greatest up-regulation with iron deficiency were *SOX4* and *Map1b. SOX4* is a transcription factor involved in the regulation of embryonic development and determination of cell fate. The protein may function in the apoptosis pathway leading to cell death and tumorigenesis. *Map1b* is involved in microtubule assembly, and plays an important role in development and function of the nervous system [35]. The two most down-regulated genes with iron deficiency were *Myo5c* and *S100a6*. *Myo5c* is involved in transferrin trafficking, and therefore plays an essential role in iron uptake and the regulation of cell proliferation. It is also likely to power actin-based membrane trafficking in a number of tissues. S100 proteins are involved in the regulation of cellular processes such as cell cycle progression and differentiation. *S100a6* may indirectly play a role in the reorganization of the actin cytoskeleton and in cell motility [36]. It was not surprising that a number of other iron metabolism genes were also down-regulated, to a lesser extent (Table S1; e.g. pirin, calreticulin, ferric-chelate reductase). Maternal iron deficiency may be expected to have a major impact upon all iron-regulated pathways in the embryo. However, we observed no change in expression of the main iron storage and transport proteins (transferrin, ferritin, transferrin receptor, hepcidin). This may indicate that the programming effects of maternal iron deficiency may not be solely or simply mediated by a gross reduction in iron supply to the developing embryo or fetus. Other mechanisms such as endocrine imbalance across the placenta and resetting of epigenetic marks are known to be involved in programming responses to undernutrition [37], [38].

The iron requirements of the day 13 embryo are likely to be small in comparison to that of the mother and may be largely met, even in the face of maternal deficiency, which may explain why no change in expression of the transport proteins was observed. Previous work by the authors determined that during pregnancy the maintenance of iron stores are prioritised towards fetal needs at the expense of the mother. It was demonstrated that despite a significant decrease in maternal liver iron content from day 0 of pregnancy in iron-deficient dams, hematocrit (Hct) levels were maintained throughout the first half of pregnancy, falling by day 21 [39]. Fetal liver iron and Hct levels measured at day 21.5 mirrored maternal concentration at the same point of gestation, i.e. they were decreased with iron-deficiency. At day 21.5 of gestation, placental and maternal liver transferrin receptor (TfR) expression was increased with iron-deficiency. An elevation in placental TfR was also found in iron-deficient day 20 placenta (FC: 3.21, P<0.03; unpublished data). Fetal liver TfR expression was unchanged by maternal iron deficiency throughout pregnancy [39], while TfR2 expression was decreased. In this study, day 13 embryos were too early in development to measure equivalent changes. Fetal transferrin expression is low in early-mid gestation and does not begin to increase until around day 18 [40].

The proteomics analysis identified only a limited number of proteins which were differentially expressed with prenatal iron restriction. This is partially due to sensitivity and methodology issues, as the separation of the proteins are limited by the pI range and size of gel. It may also be due to post translational modifications allowing a range of spots for a protein, therefore diluting the potentially significant changes in expression. Despite these limitations, there was a remarkable similarity between the microarray and proteomic results in terms of the processes and pathways affected. These proteins could be broadly categorised by function, including cytoskeletal remodelling, cell proliferation and the proteasome complex, which were also identified by the earlier gatekeeper protein analysis [24]. However, the actual proteins with significantly differing expression altered between the two studies. For example, actin-related protein 3 and tubulin α-1 chain were key gatekeeper proteins associated with cytoskeletal functions, whereas ADP-ribosylation factor-like 3, dihydropyrimidinase-related protein 2 and chaperonin containing TCP1 were identified in this role with iron deficiency. Only SUG1, a subunit of the proteasome complex, featured as a significantly affected protein in both studies. This emphasises the importance of the complex, which is related to metabolic regulation and cell cycle progression, in nutritional studies.

A small number of proteins were also significantly differentially regulated in the same direction at the gene level as shown by the microarray. Further processes which the proteomics highlighted in this study were protein folding, unfolding and transport. Failure of normal folding, accumulation of denatured proteins or failure of the proteolytic machinery of a cell can lead to a build up of potentially damaging polypeptides which could cause cellular dysfunction or trigger apoptosis. The proteasome complex and molecular chaperones function together as a quality-control system to selectively eliminate abnormal proteins. A number of chaperone proteins that were down-regulated following exposure to a prenatal FeD diet are involved in folding of cytoskeletal components (chaperonin) and other targets of interest such as p53 (nucleophosmin).

As this study used whole embryos, the gene and protein changes noted are expressed within a heterogenous cell population. Therefore a limitation of this study is that it cannot be concluded that impacted processes are related specifically to development of any specific organs, systems or tissues. Further work will be needed to isolate the location of the key genes and proteins affected by iron deficiency in tissues of interest, such as the kidney. The dilution effect conferred by using whole embryos may be the reason for the modest fold-change values found in this study, and for generalised processes such as cell division being the most highly impacted by the dietary insult, rather than tissue-specific effects. As considered in our previous study [24], criteria concerning the validation of microarray techniques are still being addressed by investigators in the field [41], [42]. However, we are satisfied that this study was adequately powered and controlled to reveal only robust results. Importantly these findings corroborate earlier observations as well as changes in protein expression in the current study.

Iron deficiency is the greatest micronutrient deficiency among humans, impacting on pregnant women in both developed and developing countries. Iron is essential for a variety of metabolic processes, and in the embryo clearly plays a key role in cellular proliferation and regulating cell-cycle proteins. This thorough study has shown that maternal iron deficiency impacts significantly on genes and proteins which regulate cell proliferation, differentiation and apoptosis in the embryo. These findings may provide important indicators of the primary mechanisms which link fetal exposure to maternal undernutrition to the development of cardiovascular, renal and metabolic disorders later in life.

## Supporting Information

Table S1 **Summary of genes which changed statistically significantly in expression in response to the iron deficient diet.**
(XLSX)
